# Establishing a model workplace tobacco cessation program in India

**DOI:** 10.4103/0019-5278.55129

**Published:** 2009-08

**Authors:** Gauravi A. Mishra, Surendra S. Shastri, Pallavi A. Uplap, Parishi V. Majmudar, Pallavi S. Rane, Subhadra D. Gupta

**Affiliations:** Department of Preventive Oncology, Tata Memorial Hospital, India

**Keywords:** Tobacco cessation, workplace, focus group discussions, oral screening, health awareness

## Abstract

**Background:**

Tobacco use is highly prevalent and culturally accepted in rural Maharashtra, India.

**Aims:**

To study the knowledge, attitude, and practices (KAP) regarding tobacco consumption, identify reasons for initiation and continuation of tobacco use, identify prevalence of tobacco consumption and its relation with different precancerous lesions, provide professional help for quitting tobacco, and develop local manpower for tobacco cessation activities.

**Settings, Design, Methods and Material:**

The present study was conducted for one year in a chemical industrial unit in Ratnagiri district. All employees (104) were interviewed and screened for oral neoplasia. Their socio-demographic features, habits, awareness levels etc. were recorded. Active intervention in the form of awareness lectures, focus group discussions, one-to-one counseling and, if needed, pharmacotherapy was offered to the tobacco users.

**Results:**

All employees actively participated in the program. Overall, 48.08% of the employees were found to use tobacco, among which the smokeless forms were predominant. Peer pressure and pleasure were the main reasons for initiation of tobacco consumption, and the belief that, though injurious, it would not harm them, avoiding physical discomfort on quitting and relieving stress were important factors for continuation of the habit. Employees had poor knowledge regarding the ill-effects of tobacco. 40% of tobacco users had oral precancerous lesions, which were predominant in employees consuming smokeless forms of tobacco.

**Conclusions:**

Identifying reasons for initiation and continuation of tobacco consumption along with baseline assessment of knowledge, attitudes, and practices regarding tobacco use, are important in formulating strategies for a comprehensive workplace tobacco cessation program.

## INTRODUCTION

Tobacco is one of the leading causes of disease and death in the world. It is responsible for a range of respiratory, cardiovascular, and reproductive tract disorders in addition to cancer of different sites in the body. In India, tobacco consumption is widely prevalent and culturally accepted. India has the highest number of oral cancer cases in the world, with tobacco being popular in smokeless forms as well. It is estimated that in the year 2005, 1,43,963 Indians would have been diagnosed with oral and pharyngeal cancers and 91,029 would have died of the disease.[1]

A tobacco-free policy at work protects nonsmokers from the harmful effects of tobacco smoke. The tobacco user receives positive peer influence from colleagues. As a large part of the day is spent at work, such a policy would help in reducing the frequency of tobacco use. This, however, may not lead to tobacco cessation among tobacco users in the absence of any support for quitting, as tobacco is highly addictive.

The department of Preventive Oncology at the Tata Memorial Hospital (TMH) initiated a workplace tobacco cessation program, considering the several advantages it has over a clinic-based setup. The workplace gives an opportunity to interact with a large number of people simultaneously, study tobacco-related work culture, and provides a stable population for follow-up. Some of the studies from western countries have analyzed the effect of smoke-free policies and different smoking cessation strategies at the workplace. We went a step further, and included both smoking as well as smokeless forms of tobacco in this workplace tobacco cessation program, which was more appropriate for the Indian setting.

The slogan for the World No Tobacco Day 2007 was “Smoke-Free Inside' which focused on making workplaces smoke-free. Our efforts went hand in hand to support the theme and a tobacco cessation program was initiated at the workplace in a chemical industry in rural Maharashtra, India. According to the Tata Memorial Hospital Cancer Registry, the majority of the men and women seeking treatment from Ratnagiri district, Maharashtra suffer from oral cancers. Data generated through the TMH community-based, rural outreach program also suggests a very high incidence of tobacco consumption in this region. Hence, one of the chemical industrial units in this area was selected to implement the workplace tobacco cessation program. Approval for the trial was received through the Scientific and Ethics Committee of the Tata Memorial Hospital and the trial was registered with clinicaltrials.gov with the registration number 458.

This program, which commenced on the World No Tobacco Day 2007, was planned for duration of one year. The long-term objective of this initiative was to formulate a Model Workplace Tobacco Cessation Program which could be replicated in other workplaces to promote tobacco control activities. The program aimed at studying the prevalence of tobacco consumption in its various forms among industrial employees and to provide professional help for quitting tobacco. The other objectives were to identify reasons for initiation and continuation of tobacco consumption habit, to assess the pre-intervention Knowledge, Attitude, and Practices (KAP) regarding tobacco consumption, compare with the post-intervention responses, and develop local manpower for tobacco cessation activities. In addition, the employees of the selected industry were screened for oral neoplasia and their findings were correlated with their tobacco habit. The present paper describes the detailed methodology and the initial findings. This will be followed another paper with the follow-up interventions and the overall results.

## MATERIALS AND METHODS

This is an interventional cohort study of one year's duration among 104 employees working in a chemical industrial unit at Ratnagiri district in Maharashtra, India.

### Inclusion and Exclusion Criteria

All 104 employees working in the selected chemical industrial unit were eligible to participate. There were no exclusion criteria.

### Program commencement

The program was conducted with due permission and support from the management, union, and employees of the selected industry. The employees of the industry were offered an introductory session on the proposed tobacco cessation and oral cancer screening program. The aim and purpose of the program were explained and the employees were invited to participate. The program was inaugurated on 31^st^ May 2007, the World No Tobacco Day, at the industrial unit. On the day prior to this, all employees took a pledge not to consume any form of tobacco on the 31^st^ of May.

### Enrollment of participants

Employees belonging to the industrial unit and who were willing to participate, were enrolled after signing the written informed consent form which was made available in English and also in the local language (Marathi).

**Figure 1:**
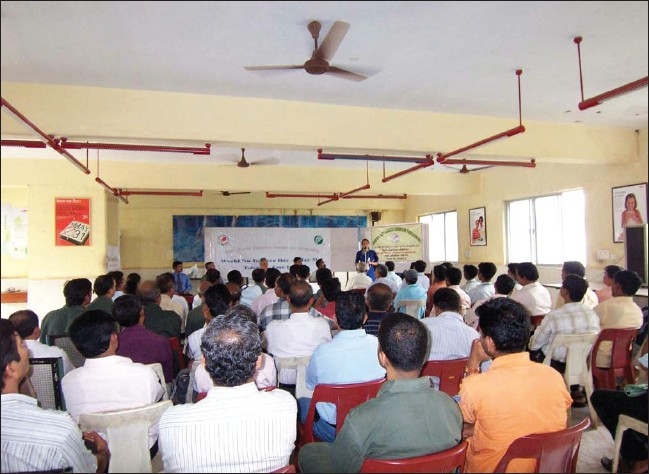
Health awareness lecture

The first session included an introductory lecture [Figure 1] and interviews of the employees to collect the pre-intervention data about various socio-demographic and risk factor variables. Diary cards in which the tobacco users were asked to record their daily tobacco consumption were introduced. All employees with or without tobacco habit were screened for oral neoplasia by naked eye visual inspection conducted by doctors. Visual inspection of the oral cavity is simple and acceptable[2,3] with a sensitivity ranging from 57.7–64%[4–6] in previous studies and a specificity ranging from 98.6 to 98.8%.[4,7] All employees using tobacco in smoking forms, were offered a smoke check by a hand-held, battery-operated device to measure the concentration of carbon monoxide in their breath. This easily operable instrument, though a good educational tool as the results are color-coded, has limited sensitivity and specificity and is unable to detect the use of smokeless tobacco products.[8]

**Figure 2:**
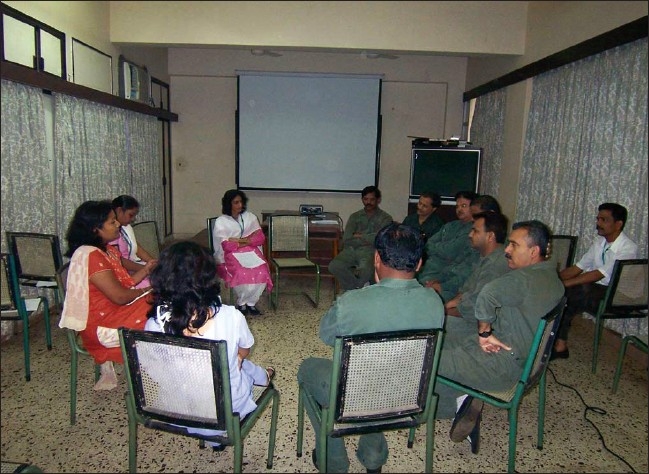
Focus Group Discussions

The follow-up sessions were offered at an interval of six to eight weeks. During these sessions, professional help in the form of health awareness sessions, focus group discussion [Figure 2], one-to-one counseling, and pharmacotherapy was provided to the employees by a team of doctors and counselors from Tata Memorial Hospital. Self-reporting of tobacco history was validated with biochemical tests.

### Capacity building

Medical and Nursing staff from the industrial medical unit and doctors and medical social workers from a local referral hospital were invited to participate as trainees during every active intervention session. This helped in local manpower development for future tobacco cessation activities in the local area. These trainees were also invited to participate in the tobacco cessation training workshop at TMH.

The data was computerized at the TMH. The socio-demographic characteristics and pre-intervention knowledge, attitude, and practices regarding tobacco use were analyzed among tobacco users and nonusers, and the groups were compared using a nonparametric test. The distribution of the overall prevalence of smoking and smokeless forms of tobacco use was calculated. The relationship of tobacco use with oral lesions and reasons for initiation and continuation of the tobacco habit were analyzed.

This trial has received institutional IRB approval and is registered with ClinicalTrials.gov.

## RESULTS

**Figure 3:**
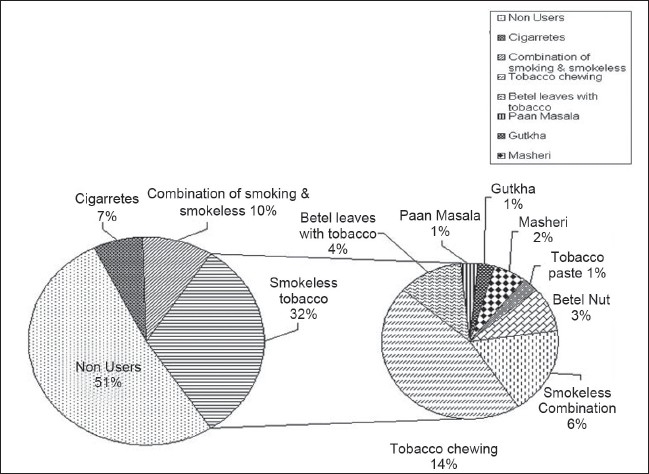
Prevalence of different forms of tobacco use

The program has been implemented for the duration of one year starting from 31^st^ May 2007 and the results are very encouraging. All employees participated in the initial interviews and oral cancer screening. Among the 104 employees of the industry, 50 (48.08%) were current tobacco users. The different forms of tobacco used by employees are shown in Figure 3. Non-smoking forms of tobacco were predominant in the employees. Tobacco chewing was the most common form of non-smoking tobacco use. Fifty-four employees (51.92%) had never used tobacco. The differences in various socio-demographic characteristics between tobacco users and nonusers are shown in Table 1. There was no difference between tobacco users and nonusers with respect to age, education, income, religion, duration of service, and the presence or absence of shift duty.

**Table 1:** Socio-demographic Characteristics of Participants In the Tobacco Cessation Program

Variables Total		Total Participants (%) 104	Tobacco non users (%) 54	Tobacco Users (%) 50	*P* Value
Age Groups (in years)	≤ 30	2 (1.92)	2 (3.7)	-	
	31–35	-	-	-	
	36–40	19 (18.27)	9 (16.67)	10 (20.00)	χ^2^ = 2.2567
	41–45	47 (45.19)	24 (44.44)	23 (46.00)	*P*=0.689
	46–50	24 (23.08)	12 (22.22)	12 (24.00)	
	> 50	12 (11.54)	7 (12.96)	5 (10.00)	
Mean Age in years (SD)	43.28 (4.58)	43.5 (5.09)	44.3 (3.97)		
Education	Primary [1–4]	2 (1.92)	0 --	2 (4.00)	
	Secondary [5–10]	14 (13.46)	9 (16.67)	5 (10.00)	
	Jr. College [11–12]	19 (18.27)	8 (14.81)	11 (22.00)	χ^2^ = 3.843
	Sr. College [13–15]	35 (33.66)	19 (35.19)	16 (32.00)	*P*=0.428
	Graduates and above	34 (32.69)	18 (33.33)	16 (32.00)	
Income per month	Rs. 10,000–20,000	30 (29.13)	16 (30.19)	14 (28.00)	
	Rs. 21,000–30,000	46 (44.66)	23 ((43.40)	23 (46.00)	χ^2^=1.0205
	Rs.31,000–40,000	19 (18.44)	11 (20.75)	8 (16.00)	*P*=0.796
	> Rs.40,000	8 (7.77)	3 (5.66)	5 (10.00)	
Religion	Hindu	102 (98.08)	54 (100)	48 (96.00)	χ^2^=2.202
	Muslim	1 (0.96)	-- --	1 (2.00)	*P*=0.332
	Others	1 (0.96)	-- --	1 (2.00)	
Duration of Service (in years)	< 5	2 (1.92)	2 (3.70)	0 --	
	5–10	1 (0.96)		1 (2.00)	
	11–15	8 (7.70)	6 (11.11)	2 (4.00)	χ^2^=6.1608
	16–20	45 (43.27)	25 (46.30)	20 (40.00)	*P*=0.187
	21–25	48 (46.15)	21 (38.89)	27 (54.00)	
	> 25	--	--	--	
Presence of Shift Duty	Yes	65 (62.50)	33 (61.11)	32 (64.00)	χ^2^=0.0924
	No	39 (37.50)	21 (38.89)	18 (36.00)	*P*=0.761

The responses of tobacco users and nonusers regarding the knowledge of harmful effects resulting from tobacco use are shown in Table 2.

**Table 2:** Comparison of Pre-Intervention responses regarding harmful effects of tobacco in tobacco users and nonusers

Knowledge-based questions on tobacco Total Participants (104)	Employees who identified	Nontobacco users (%) the risk correctly (%)	Tobacco users (%)	*P* Value
Employees who identified tobacco as injurious to health	104 (100)	54 (51.92)	50 (48.08)	--
Employees who identified tobacco as risk factor for Cancer	99 (95.19)	53 (53.54)	46 (46.46)	χ^2^ =2.144 (*P*=0.143)
Employees who identified tobacco as risk factor for Bronchitis	41 (39.42)	21 (51.22)	20 (48.78)	χ^2^=0.0134 (*P*=0.908)
Employees who identified tobacco as risk factor for Heart Attack	49 (47.16)	24 (48.98)	25 (51.02)	χ^2^=0.3216 (*P*=0.571)
Employees who identified tobacco as risk factor for Paralysis/Stroke	41 (39.42)	21 (51.22)	20 (48.78)	χ^2^= 0.0134 (*P*=0.908)
Employees who identified tobacco as risk factor for wrinkling of skin, early aging, infertility in females and impotence among males	58 (55.77)	29 (50.00)	29 (50.00)	χ^2^=2.517 (*P*=0.284)
Employees who identified cigarettes, beedi, tobacco chewing, betel nuts, betel leaves with tobacco, paan masala, gutkha, hookah all as harmful	45 (43.27)	24 (53.33)	21 (46.67)	χ^2^=0.0632 (*P*=0.802)
Employees who identified filtered, low tar, low nicotine cigarettes as unsafe	57 (54.81)	28 (49.12)	29 (50.88)	χ^2^=0.396 (*P*=0.529)
Employees who identified passive smoking dangerous	101 (97.12)	54 (53.47)	47 (46.53)	χ^2^=3.336 (v=0.068)
Employees who knew that tobacco is dangerous even when consumed infrequently	86 (82.69)	45 (52.33)	41 (47.67)	χ^2^=0.0322 (*P*=0.857)
Employees who knew that professional help is available to quit tobacco	78 (75)	36 (46.15)	42 (53.85)	χ^2^=4.160 (*P*=0.041)

Many tobacco users were aware that tobacco was harmful, however, they were unsure about the intensity and type of damage that it caused. Although, 100% of the employees agreed that tobacco was injurious to health, only 37 employees (35.58%) knew all the ill effects of tobacco. The majority were only aware that tobacco caused cancer. Most (97%) employees identified passive smoking as being dangerous for health and 83% of the employees knew that tobacco was dangerous even when consumed infrequently. There was a significant difference in the knowledge regarding availability of professional help for quitting tobacco, with tobacco users being more aware of this fact. The employees had mainly received the information about harmful effects of tobacco from printed media, hoardings, television, the industry physician, and their family physicians.

**Figure 4:**
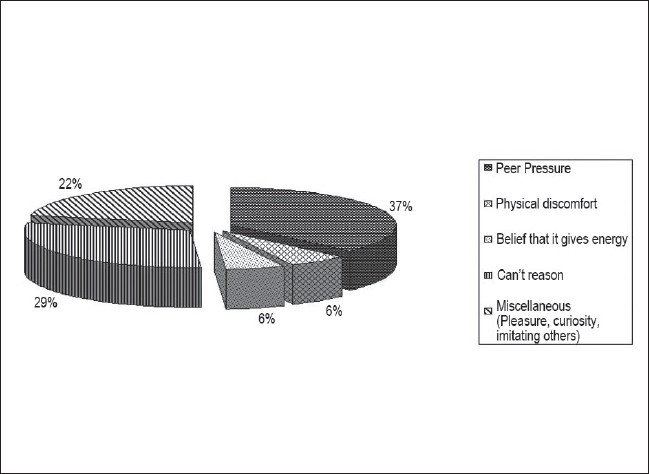
Factors responsible for initiation of tobacco habit

**Figure 5:**
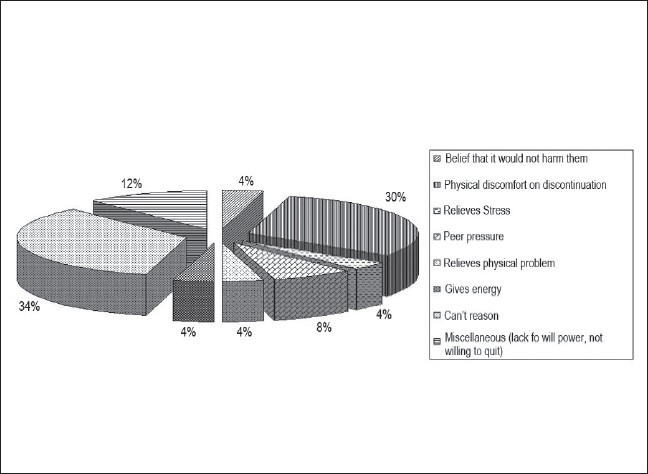
Factors responsible for continuation of tobacco habit

On an average, the tobacco users spent Rs. 66.65 (1.39 USD), (Rs. 48 = 1 USD) every month on tobacco; among which, the employees using smoking forms were spending Rs. 293.33 (6.11 USD) per month, the employees using non-smoking forms spent Rs. 22 (0.46 USD) per month, and the employees using a combination of smoking and non-smoking forms spent Rs. 73.50 (1.53 USD) per month. The mean age at the initiation of cigarette smoking was 24.65 years and at the initiation of the smokeless tobacco habit was 27.51 years. The main reasons for the initiation of the tobacco habit by tobacco users [Figure 4] were peer pressure, miscellaneous factors like pleasure and imitation of others. The main reasons for continuation of tobacco use (Figure 5) were the belief that it would not harm them personally, to avoid physical discomfort on quitting, and to relieve stress. Fifteen employees (10.71%), of whom ten (20%) were tobacco users and five (9.26%) were nonusers had some family member, mainly siblings and parents, with the tobacco habit.

On oral examination, 20 employees were found to have oral precancerous lesions (18 had leukoplakias and two erythroplakias). The majority of the lesions were seen among employees using non-smoking forms of tobacco (two lesions among employees with smoking forms, 13 in employees with non-smoking forms, and five among employees using a combination). Among the 17 employees using smoking forms of tobacco, 13 employees had a Fagerstorm score of zero. Among 43 employees using non-smoking forms of tobacco, 32 employees had a Fagerstorm score of 4 and above. More lesions (80%) were seen among employees with a Fagerstorm score of 4 and above and using smokeless form of tobacco. Other medical illnesses had nearly an equal distribution among the tobacco users and nonusers.

## DISCUSSION

India is the second largest consumer of tobacco in the world, where tobacco is popular in smokeless forms as well. Literature on tobacco cessation is mostly from the western countries, which made it imperative to study it in the Indian context. The industrial unit described here had a No Tobacco Policy on its campus for over a period of 30 years, in spite of which a very high prevalence of tobacco consumption (48%) was recorded, indicating that tobacco-free workplaces may not result in tobacco cessation without any support for quitting. Tobacco use was mainly in the nonsmoking forms, reflecting the cultural practices of the community. In a study conducted among Indian men, 69.3% were found to be current tobacco users, among whom 23.6% were smokers.[9] In the Kerala community study, 72% of men and 6% of women were “ever users,” i.e., had used tobacco at least once in their lives.[10] In Nepal, the overall prevalence of ‘ever users’ of tobacco products was 13.9% and of ‘current users’ was 10.2% among junior college students.[11]

The participation in the first session of interviews and the initial screen was 100%, indicating that employees accept tobacco cessation activities when conducted at their workplace. No difference was found in the socio-demographic profile between the tobacco users and the nonusers. This may be attributed to the fact that the employees belonged to the same cohort with similar socio-economic and religious backgrounds. The educational level was inversely associated with tobacco use of all kinds,[9,12–14] except cigarette smoking in a study in Mumbai.[9] In a survey in India, although no significant differences were seen in the prevalence of tobacco use among Hindu, Muslim, and Christian populations, the Sikh population, however, was found to have a significantly lower prevalence of tobacco consumption.[13] Use of smokeless tobacco products was high among Buddhists and low among Christians in Mumbai, while smoking was high among Muslims and Christians and low among Buddhists.[9] Tobacco use was seen to be more commonly associated with low socio-economic status[13,14] and scheduled castes and schedules tribes[13,14] in India. Tobacco use was also found to be correlated with occupation in Mumbai, with unskilled male workers, male service workers, and unemployed individuals being more at risk than professionals.[12]

In the present study, the employees were aware that use of tobacco including passive smoking was harmful, however, they could link only a few diseases like cancer to it. Most employees were unaware about the risk of tobacco on the respiratory and cardiovascular systems. Printed media, television, and doctors were the main source of this health information. Most students in Jaipur schools were aware that tobacco use was harmful and associated it as a major risk factor for respiratory diseases.[15] In rural Kerala, 96.6% of the participants knew that tobacco use was harmful for health, however, only 22.5% of the participants knew that it caused cardiovascular diseases.[16] Electronic and print media were the common source of this knowledge; only 20.6% subjects reported that health care workers were a source of such knowledge.[16] In Italy, the knowledge of risk associated with smoking was significantly higher in more educated subjects and in past smokers compared to current smokers.[17] According to an international tobacco survey in four countries, about 10% or more of smokers did not believe that smoking caused heart disease. Over 20 and 40% did not believe that smoking caused stroke and impotence, respectively. Higher education and income were associated with higher awareness.[18]

The average monthly expenditure on tobacco use in the current study's participants is Rs. 66.65 (1.39 USD), with employees using smoking forms spending significantly higher amounts as compared to the employees using smokeless forms. In a study in Nepal, the average daily expenditure on tobacco was 20 Nepalese rupees (~0.3 USD).[11] In Tehran, 41.8% of the population had daily smoking expenses of 2,510 to 4,500 Rials (1 Dollar = 9000 Rials) while the mean was 4,680 ± 388.78 Rials.[19]

The mean age at initiation of cigarette smoking was 24.65 years and at initiation of chewing tobacco was 27.51 years among these industrial unit employees. Survey data in Kerala suggest that the age at initiation of tobacco use appears to be falling.[10] The median ages at initiation of cigarette smoking and chewing tobacco were 16 and 15 years respectively among junior college students in Nepal.[11] The mean age of initiation of smoking was 21 ± 8.19 years in Tehran.[19]

Among the tobacco users in the present group of participants, peer pressure, pleasure and imitating others were the most important reasons for initiation of the tobacco habit, and the belief that tobacco would not harm them personally, avoidance of physical discomfort on discontinuation, and relief of stress were important reasons for continuation of the habit. Predisposing, reinforcing, and enabling factors associated with tobacco use behaviors among Cambodian Americans included peer group influences, smoking adopted as a coping method, tobacco used for medicinal purposes, and smoking practised within cultural traditions.[20] In the present study, 20% of tobacco users as opposed to only 9% of nonusers had some family member who consumed tobacco, suggesting that the tobacco habit is more common in families with addiction. In Jaipur, tobacco use was significantly more common in families of children who used tobacco.[15] The tobacco use habits of fathers and peers are significant influences on youth smoking.[10] Family members, teachers, and friends using tobacco products were correlated with tobacco use in Nepal.[11]

There is increasing and indisputable scientific evidence showing that tobacco is a major cause of chronic bronchitis, emphysema, and lung cancer, as well as a major risk factor for myocardial infarction, certain pregnancy-related and neonatal disorders, and a number of other serious health problems. It also has harmful effects on those who are involuntarily exposed to tobacco smoke.[21] In the present study, however, medical illnesses were equally distributed among the tobacco users and nonusers. In Helena, Montana, a study found that during the six months that the law that prohibited smoking in most workplaces was enforced, the number of admissions for acute myocardial infarction fell significantly.[22]

In the present study, 40% of tobacco users (19.23% of total employees) had oral precancerous lesions. The prevalence of oral precancerous lesions was more among tobacco chewers than among smokers. The severity of the lesion correlated well with the severity of addiction on the Fagerstorm score. The results of the Kerala study suggest that tobacco chewing is the most important risk factor for multiple, oral, premalignant lesions and dose-response relationships were seen for the frequency and duration of tobacco chewing with the risk of multiple, oral, premalignant lesions.[23]

## CONCLUSIONS

This paper reflects the tobacco consumption practices among industrial employees in rural India. The unique feature of this program is that it aims at tobacco cessation and not just smoking cessation, a very important aspect for countries like India, where nonsmoking forms of tobacco use are predominant. Local manpower development for tobacco cessation was inbuilt within the program, to ensure continuation of assistance to the employees even after the study was complete, and to encourage similar activities in nearby industries. A very high rate of oral precancerous lesions detected for the first time among the employees, suggests that in spite of having good medical facilities, the employees are hardly ever examined for oral neoplasia. High tobacco consumption rates among employees of the current industrial unit with the No Tobacco policy indicates that tobacco cessation and awareness should be integrated within industrial health guidelines.
